# 

**Published:** 1857-06

**Authors:** 


					﻿VOL. IV.]
[NUMBER 1.
MAY AND JUNE, 1857.
IOWA MEDICAL JOURNAL.
CONDUCTED BY THE FACULTY OF THE
MEDICAL DEPARTMENT
s
S
£
I a
K
. X
l 2
' kJ
£
£
o
u
o
f
£
$
S
£
s
I
0
OF THE
IOWA STATE UNIVER siTy
editobs:
J. C. HUGHES, M. D.,
Prof, of Surgery in the Medical Department of the Iowa UnWeraioy
WELLS R. MARSH, M. D.
Prof, of Chemistry and Toxicology in Med. Dwp't. of Iowa Uni»ersity
KEOKUK, IOWA:
PRINTED AT THE TIMES BOOK AND JOB OFFICE-
1857.
				

